# Institutional Questions

**Published:** 1899-11-18

**Authors:** 


### INSTITUTIONAL QUESTIONS.

### Hospital Laundries.

"A Matron" writes: I should be glad of some practical hints upon the management of a hospital laundry. Is there any book on the subject which would help one in starting a laundry on a small scale?

*** We do not know of any book dealing exclusively with hospital laundries. A book on "Laundry Management," by the editor of the *Laundry Journal*, is published by Crosby Lockwood and Son, price 2s. 6d., which might help you, and there is a chapter on "The Hospital Laundry" in an American book, "Handbook for Hospitals," an extremely useful little publication, to be had from Putnam's Sons, Bedford Street, Strand. You would find it well to visit some of the most modern of existing hospital laundries, where you would pick up more useful information in an hour than by the study of many books on the subject. The laundry department at Guy's Hospital is thoroughly up-to-date, and is well worth a visit.

